# Widely spaced and divergent inverted repeats become a potent source of chromosomal rearrangements in long single-stranded DNA regions

**DOI:** 10.1093/nar/gkad153

**Published:** 2023-03-15

**Authors:** Anissia Ait Saada, Wenying Guo, Alex B Costa, Jiaxin Yang, Jianrong Wang, Kirill S Lobachev

**Affiliations:** School of Biological Sciences and Institute for Bioengineering and Bioscience, Georgia Institute of Technology, Atlanta, GA 30332, USA; School of Biological Sciences and Institute for Bioengineering and Bioscience, Georgia Institute of Technology, Atlanta, GA 30332, USA; School of Biological Sciences and Institute for Bioengineering and Bioscience, Georgia Institute of Technology, Atlanta, GA 30332, USA; Department of Computational Mathematics, Science and Engineering, Michigan State University, East Lansing, MI 48824, USA; Department of Computational Mathematics, Science and Engineering, Michigan State University, East Lansing, MI 48824, USA; School of Biological Sciences and Institute for Bioengineering and Bioscience, Georgia Institute of Technology, Atlanta, GA 30332, USA

## Abstract

DNA inverted repeats (IRs) are widespread across many eukaryotic genomes. Their ability to form stable hairpin/cruciform secondary structures is causative in triggering chromosome instability leading to several human diseases. Distance and sequence divergence between IRs are inversely correlated with their ability to induce gross chromosomal rearrangements (GCRs) because of a lesser probability of secondary structure formation and chromosomal breakage. In this study, we demonstrate that structural parameters that normally constrain the instability of IRs are overcome when the repeats interact in single-stranded DNA (ssDNA). We established a system in budding yeast whereby >73 kb of ssDNA can be formed in *cdc13-707fs* mutants. We found that in ssDNA, 12 bp or 30 kb spaced *Alu*-IRs show similarly high levels of GCRs, while heterology only beyond 25% suppresses IR-induced instability. Mechanistically, rearrangements arise after *cis*-interaction of IRs leading to a DNA fold-back and the formation of a dicentric chromosome, which requires Rad52/Rad59 for IR annealing as well as Rad1-Rad10, Slx4, Msh2/Msh3 and Saw1 proteins for nonhomologous tail removal. Importantly, using structural characteristics rendering IRs permissive to DNA fold-back in yeast, we found that ssDNA regions mapped in cancer genomes contain a substantial number of potentially interacting and unstable IRs.

## INTRODUCTION

Mutations and chromosomal rearrangements are footprints of genetic instability, characteristically associated with genetic disorders but necessary for evolution and genetic diversity. Sources of genetic instability are diverse and can be either endogenous or exogenous in nature. The architecture of the genome itself is very important for genomic stability. Indeed, certain chromosomal sites, composed of fragile DNA motifs that can adopt secondary (non-B) DNA structures, are hotspots for instability (1). Among them, inverted DNA repeats (IRs), also called palindromic sequences, are found in the genomes of both prokaryotes and eukaryotes and are associated with the occurrence of several human diseases such as male infertility, developmental disorders and cancer (2–17). Due to their intrinsic propensity to form hairpins and cruciforms, long IRs are predisposed to double-strand break formation, fork stalling, allelic and non-allelic recombination, and gross chromosomal rearrangements (GCRs) in several organisms (examples in (18–33).

Under normal DNA metabolism, in dsDNA, the deduced mechanism for IR instability stems from cruciform extrusion that triggers double strand breaks. Therefore, the ability of IRs to induce recombination and chromosomal rearrangements is inversely correlated with the length of the intervening sequence separating the two arms of the IR and the degree of sequence divergence since such changes in both structural parameters substantially lessen the likelihood of cruciform extrusion in dsDNA (34–37). For example, an increase in the spacer from 12 to 20 bp causes a ∼40-fold reduction in *Alu*-IR-induced recombination. Similarly, an increase in IR divergency leads to an exponential decrease in recombination and a complete loss of IR instability when homology is lowered to 75%. Even a single mismatch located proximal to the center of symmetry significantly reduces the recombinational potential of *Alu*-IRs (34). In contrast, ssDNA formation renders the intramolecular interaction of IRs more likely. Long stretches of deleterious ssDNA can form during replication stress and uncoupling of leading and lagging strand synthesis, DNA resection of DSBs or uncapped telomeres, and break-induced replication (BIR) (reviewed in (38)). A genome-wide screen performed in budding yeast, revealed that defects in the replication machinery increase the fragility potential of IRs, likely because replication-defective mutants are prone to ssDNA accumulation which facilitates hairpin formation (25). In the same line of evidence, the presence of an IR in resected DNA during DSB repair or BIR favors intra-strand annealing between repeats, leading to inverted dimer formation or IR deletion, respectively (39–44).

An under-investigated question is: to what extent do imperfect IRs interact in ssDNA? One way of generating ssDNA on demand is via controllable induction of telomere uncapping in CST (Cdc13-Stn1-Ten1) complex mutants (45–49). For example, in budding yeast, expression of the thermosensitive allele *cdc13-1* leads to extensive 5′-3′ DNA resection, mostly mediated by Exo1, and checkpoint activation (47,48,50,51). Defects in the CST complex in budding yeast have been associated with an increase in chromosomal instability induced by fragile DNA motifs located 40–74 kb away from the telomere such as *Alu* quasi-palindromes and triplex-forming GAA/TTC trinucleotide tracts (52,53). Spivakovsky-Gonzalez *et al.* recently reported that GAA/TTC instability is increased in the *cdc13-1* mutant in an Exo1-dependent manner (53). However, the mechanisms of secondary-structure-forming motif instability upon CST deficiency are not very well understood.

Here, we took advantage of the *cdc13-707fs* mutant we isolated to systematically study the impact of IRs with long spacers and different degrees of homology on genome instability in the context of ssDNA. Using a *CAN1* reporter, we found that mutagenesis, which is reflective of ssDNA formation, can reach up to 73 kb away from the telomere. Importantly, we found that distantly spaced and heterologous *Alu* IRs contained within ssDNA, as opposed to dsDNA, have a drastically different potential to induce chromosome instability independently of their ability to induce DSBs. Mechanistically, we show that the *cis*-interaction of IRs leads to a DNA fold-back that subsequently leads to dicentric chromosome formation, breakage and GCRs. We found that rearrangements induced by ssDNA-containing IRs require the single strand annealing proteins Rad52 and Rad59 and the 3′ nonhomologous flap removal complex Rad1-Rad10-Slx4 with its modulating factors, Msh2-Msh3 and Saw1. Since we found that decreasing the degree of homology to 75% or increasing the spacer length up to 30 kb had little or no impact on the ability to induce instability, we applied these parameters to map potentially unstable IRs in the human genome in transiently single-stranded regions as indicated by APOBEC-induced mutation clusters (reviewed in (38)). Our data show that, in addition to being extremely mutagenic, ssDNA can be highly deleterious because it favors interaction between significantly distant and divergent inverted repeats, which previously were not considered as triggers of genome instability.

## MATERIAL AND METHODS

### Strains, plasmids and oligonucleotides

Yeast strains and oligonucleotides used in this study are listed in Supplementary Tables S6 and S7. The strains used in this study are isogenic and based on MATα, *bar1*Δ, *his7-2*, *trp1*Δ, *ura3*Δ, *leu2-3 112, ade2*Δ, *lys2*Δ, *cup1*Δ, *yhr054c*Δ, *cup2*Δ, *V34205::ADE2, lys2::IR, V29616::CUP1*. The GCR cassette composed of the counter selectable markers *CUP1*, *CAN1* and *ADE2* is located on the left arm of chromosome V. IRs with a <10 kb spacer are located within *LYS2*, which is telomere-distal to the GCR cassette. For all experiments, freshly thawed yeast strains were used. All *cdc13-707fs* strains were grown at 23°C and then shifted to 30°C. The media used in this study are YPD (1% yeast extract, 2% peptone, 2% dextrose) and arginine drop-out synthetic medium supplemented with 60 mg/L of canavanine and 4 mg/L (low amount) of adenine. The *RAD52, RAD59, SLX4*, *RAD1*, *MSH2*, *MSH3* and *SAW1* genes were disrupted using one of the following markers: *TRP1*, *kanMX4* (G418), *hphMX* (hygromycin) or *natMX* (nourseothricin). The carboxy-terminal part of Ddc1 was fused to yEGFP at its endogenous location using the plasmid pKT127 (Euroscarf P30175). To estimate the rate of mutagenesis at different distances (55, 48, 73 and 92 kb) from the left arm of chromosome V, the original *CAN1* gene was deleted by the *TRP1* marker and the *CAN1-natMX* reporter cassette was integrated at the chosen location.

### Random mutagenesis and isolation of *cdc13-707fs*

To identify mutations in CST complex subunits exhibiting a hyper-GCR phenotype at *Alu*-IRs (separated by 12 bp and sharing 94% homology), the genes coding each subunit were randomly mutagenized. *CDC13*, *STN1*, and *TEN1* were individually cloned into plasmids with a pRS414 backbone and the *URA3* marker was then inserted next to each gene. The resulting plasmids were propagated in the mutator *E. coli* strain, XL-1 Red (Agilent), to generate randomly mutagenized CST subunit-containing plasmid libraries. Yeast strains with *Alu*-IRs were transformed with the mutagenized DNA fragments containing one of the *URA3*-tagged CST subunit genes. Upon selection on media lacking uracil, Ura^+^ transformants were verified for their level of IR-induced chromosome instability using the GCR assay (Narayanan *et al.*, 2006). One clone expressing a mutagenized *URA3*-tagged *CDC13* and exhibiting a high GCR level was isolated. Sequencing of *CDC13* revealed a + 1 adenine insertion in the 7A homonucleotide run spanning from nucleotide 2115 to 2121 that causes a frameshift after residue K707 and generates a stop codon 65 amino acids downstream. The allele is referred to as *cdc13-707fs*. Strains expressing this allele were recreated by transformation using the *URA3*-tagged *cdc13-707fs* obtained by polymerase chain reaction from the gDNA of the strain obtained in the screen.

### Cell survival by spot test assay

Single colonies from WT or mutated *CDC13* strains grown on YPD at 23°C were resuspended in 200 μl of water. Cells were spotted as serial 10-fold dilutions onto YPD plates and incubated at 23, 30 and 37°C for 2–3 days prior to imaging.

### Construction of *Alu*-IR strains

*Alu*-IRs-containing strains with <10 kb spacers and >65% homology were constructed as described in Lobachev *et al.* and Ait Saada *et al.* The 1.5 kb spacer corresponds to the *kanMX4* gene, the 3 kb to the *kanMX4* and *URA3* genes and the 5 and 7 kb to a sequence from the lambda phage. To ensure that the *Alu*-IRs with >10 kb spacers remain in the resection window upon telomere uncapping in *cdc13-707fs*, a modified *CAN1-ADE2*-based GCR assay was built. The *CAN1* and *ADE2* genes (forming the GCR cassette) were relocated ∼10 kb away from the telomere while *LYS2* remained at the same location (∼43 kb away from the telomere). A single *Alu* element (320 bp) with a *kanMX* marker was inserted into *LYS2*. A second *Alu* element, in the inverted orientation, along with the *URA3* marker was inserted 10, 20 or 30 kb away from the former *Alu* sequence. To ensure that the modified GCR assay works the same way as the original system, a 10 kb spacer IR was also generated by inserting a 10 kb lambda sequence into the *Alu*-IR within *lys2* using the *delitto perfetto* approach (54) as described in Ait Saada *et al.* The resulting IR is located ∼53 kb away from the telomere, and thus in resection window in *cdc13-707fs* upon telomere uncapping. The GCR rates induced by the 10 kb spacer *Alu*-IR in the strains with the original and modified GCR systems were similar (9.2 × 10^−7^ [8.5 × 10^−7^–11 × 10^−7^] versus 8.5 × 10^−7^ [7 × 10^−7^–9.7 × 10^−7^] in WT and 2.4 × 10^−4^ [1.8 × 10^−4^–2.8 × 10^−4^] versus 2.5 × 10^−4^ [1.5 × 10^−4^–3.5 × 10^−4^]. 65% *Alu*-IRs were designed and built as follows. N14 *Alu* sequence (Lobachev *et al.*) was modified by introducing 29 randomly distributed single nucleotide changes to further decrease the homology between N14 *Alu* and HS-*Alu* consensus sequences from 75% to 65%. SalI and BamHI sites were added at the 5′ and 3′ends of this modified *Alu* sequence, accordingly. The designed sequence was synthesized by Azenta Life Sciences and the SalI and BamHI fragment was inserted next to HS-*Alu* in inverted orientation in the *LYS2* gene on the integrative vector as previously described (Lobachev *et al.*). The resulting pKL679 vector was used to transfer 65% *Alu*-IRs into the chromosomal *LYS2* gene using a two-step replacement procedure.

### GCR and mutagenesis rate estimation by fluctuation test

Freshly thawed strains were grown on YPD plates at 23°C for 5 days and then plated on YPD and incubated at 30°C for 3 days to form single colonies. A minimum of 12 independent colonies per strain were selected for a fluctuation test and appropriate dilutions were plated on YPD and canavanine-containing plates (arginine drop-out synthetic medium supplemented with 60 mg/L of canavanine and a low amount, 4 mg/L, of adenine). Plates were then incubated at 23°C. White, canavanine resistant colonies reflect *CAN1* mutagenesis whereas red, canavanine resistant colonies represent GCR events (arm loss). Mutation and GCR rates were calculated using the formula μ = *f*/ln(*N*μ) as described in Drake (55). Data are represented as the median rate ± 95% confidence interval.

### UV-induced mutagenesis

Freshly thawed strains were grown on YPD plates at 23°C for 5 days and then plated on YPD and incubated at 30°C for 3 days. Single colonies were then inoculated into 10 ml of YPD overnight to reach ∼ 1–2 × 10^8^ cells/ml. Prior to UV-treatment, cells were washed, resuspended in water and transferred to Petri dishes. In a UV cross-linker (Stratalinker 2400), cell suspensions were exposed to 45 J/m^2^ UV-C. Appropriate dilutions of UV-treated or untreated cells were plated on YPD and canavanine-containing plates and incubated at 23°C. Colonies grown on canavanine-containing plates were replica plated on lysine drop-out synthetic medium to score for Can^R^Lys^−^ colonies.

### Inverted dimer detection

Single colonies grown at 23°C were patched on YPD and incubated at 30°C overnight. Cells were then inoculated in 10 ml of YPD and grown overnight at 32°C. About 2.5 × 10^9^ cells/ml were embedded in 0.5% low-melting agarose (Lonza, NuSieve™ GTG™ Agarose, in 0.1M EDTA) plugs. Plugs (the equivalent of ∼1 × 10^8^ cells) were processed as described in Ait Saada *et al.* and submitted to restriction digestion with 50 units of SgsI overnight. Digested plugs were loaded on a 1% agarose gel and run in 1X TBE for ∼20 h at 55 V. The agarose gels were treated consecutively with 0.25 N HCl, alkaline buffer (1.5 M NaCl, 0.5 M NaOH) and neutralization buffer (1.5 M NaCl, 1 M Tris, pH 7.5), and the separated DNA fragments were then transferred to a positively charged nylon membrane (Perkin Elmer NEF988001PK) in 10X SSC by capillary. Southern hybridization was performed using a ^32^P-radiolabeled, centromere-proximal *LYS2*-specific probe in PerfectHyb Plus Hybridization Buffer at 69°C overnight. The membranes were washed twice in a buffer containing 0.1X SSC and 0.1% SDS at 69°C and were exposed to a phosphor storage screen.

### Structural analysis of GCRs by whole genome sequencing

Genomic DNA extractions were prepared from *cdc13-707fs* and 12 red, canavanine-resistant colonies resulting from a GCR in *cdc13-707fs* with a YeaStar Genomic DNA Kit (Zymo Research) and were purified by ethanol precipitation. Samples were barcoded and sequencing libraries were prepared with an Oxford Nanopore Technologies (ONT) rapid barcoding kit (ONT, SQK-RBK110.96). Sequencing was performed with a MinION sequencer using a 9.4.1 flow cell (ONT). Using Guppy-CPU 6.0.1 (ONT), sequencing data were basecalled with the fast basecall model, demultiplexed and aligned to the SacCer3 reference genome (National Center for Biotechnology Information). ChrV terminal deletions and duplications were visualized in Integrative Genome Viewer (Broad Institute). Breakpoints were estimated by read depth variation and were confirmed by the presence of junction-spanning long reads, which were BLASTed against the SC288C reference genome (National Center for Biotechnology Information, accessed through Saccharomyces Genome Database) to fine-map breakpoints.

### Southern blot analysis of telomere length

Genomic DNA from WT and *cdc13-707fs* strains grown overnight at 30°C was submitted to restriction digestion by XhoI (56). XhoI-digested fragments were separated in a 0.9% agarose gel and run in 1× TBE buffer at 55 V for 17 hr. Telomeres were revealed after Southern blot hybridization (as described in the section *Inverted dimer detection*) using a ^32^P-radiolabeled, Y’ element-specific probe. The locations of the XhoI restriction sites and the probe allow the detection of both subtelomeric elements (a band of 5.5 or 6.5 kb) and telomeres (a smear around 1.3 kb in WT strains).

### Inverted TE pair distribution in ssDNA regions of cancer genomes

In this study, clustered mutation data were collected from the Supplementary Table S5 in Sakofsky *et al.*, which contains APOBEC hypermutated clusters in tumor genomes sequenced by the PanCancer Analysis of Whole Genomes (PCAWG) project (57). The transposable element (TE) annotations in hg19 were obtained from the RepeatMasker project (Smit, AFA, Hubley, R & Green, P. RepeatMasker Open-4.0. 2013–2015; http://www.repeatmasker.org). Three types of clusters were used in TE-inverted pair calling: (i) CG coord, C- or G-coordinated; (ii) coord with terminal CG, C-coordinated clusters adjacent to a single G or G-coordinated clusters adjacent to a single C; and (iii) single-switch clusters. As per the analysis in Sakofsky *et al.*, these cluster categories are generated by APOBEC cytidine deaminase(s) in long hypermutable ssDNA distributed across cancer genomes. The annotated TEs in each cluster were collected from RepeatMasker. All potential TE-inverted pairs belonging to the same TE families, which included both convergent and divergent TE pairs, were aligned using BLAST (58,59) in each cluster. The TE-inverted pairs with matching fractions >75% and matching lengths >100 bp were considered successful alignments. Only the clusters containing TE-inverted pairs with genomic distances <30 kb were kept. All TE-inverted pairs are listed according to their corresponding TE family in Supplementary Table S5 where the chromosomal location and the length of the TEs as well as the distance and homology between the TEs of the pair are indicated.

### Live cell imaging

Three independent cultures were grown overnight in filtered synthetic complete medium at 30°C. Snapshot microscopy was performed using an AxioVert 200M Zeiss microscope equipped with an AxioCam HRm camera. Cells were visualized with a 100× oil-immersion objective and images of several focal planes were taken to cover the depth of the nuclei using AxioVision Zeiss software. Exposure time was set at 6 s for the GFP channel. Images were analyzed with ImageJ software.

### Quantification and statistical analysis

***GCR and mutagenesis rate:*** All data presented are the median of the rates for at least 12 biological replicates and a 95% confidence interval is indicted (Drake, 1991).

## RESULTS

### Generation of ssDNA reaches up to 73 kb in the *cdc13-707fs* mutant

In a genome-wide screen aiming to identify factors crucial for maintaining IR stability, we found that down-regulation of *CDC13* induces a 3-fold increase in chromosomal fragility at 12 bp spaced 100 and 94% homologous *Alu* quasi-palindromes (25). To understand how a defect in the CST complex leads to *Alu*-IR instability, the *CDC13* gene was randomly mutagenized to identify an allele exhibiting a hyper fragility phenotype for a 94% homologous *Alu*-IR located ∼43 kb away from the telomere. We isolated a hyper fragile mutant harboring a + 1A insertion in the natural 7A homonucleotide run spanning from 2115 to 2121 nts at the C-terminus of the gene. This insertion causes a frameshift after K707 and generates a stop codon at the 772nd amino acid, in the OB4-fold domain. It is likely that this mutation, named *cdc13-707fs*, weakens the interaction between Cdc13 and Stn1 (60).

Defective Cdc13 proteins are usually thermolabile and are deficient in telomere capping at nonpermissive temperatures, which disturbs telomere length homeostasis. Growth of the commonly used *cdc13-1* mutant is strongly impaired at temperatures higher than 26°C (46). We analyzed the temperature sensitivity of *cdc13-707fs* and found that, similar to *cdc13-1*, *cdc13-707fs* is thermo-sensitive at 37°C and the growth defect at high temperatures is Exo1 dependent. However, in contrast to *cdc13-1*, *cdc13-707fs* does not show a significant decrease in viability or any growth defect at 30°C (Supplementary Figure S1A). The hyper-GCR phenotype in *cdc13-707fs* strains with the 12 bp spacer *Alu*-IR is only observed when cells are grown at the semi-permissive temperature (Supplementary Figure S1B). In addition, *cdc13*-*707fs* mutants at 30°C have extended telomeres (Supplementary Figure S1C), a phenotype often associated with CST dysfunction in telomere maintenance (61). In agreement with telomere uncapping and ssDNA formation, ∼24% of *cdc13-707fs* cells exhibit Ddc1-GFP foci at the semi-permissive temperature 30°C (Supplementary Figure S1D), which is indicative of checkpoint activation (62). This makes *cdc13-707fs* a useful tool for studying telomere capping deficiency at 30°C without impairing cell growth and inducing cell death. Hence, all the following experiments were performed at 30°C.

**Figure 1.**
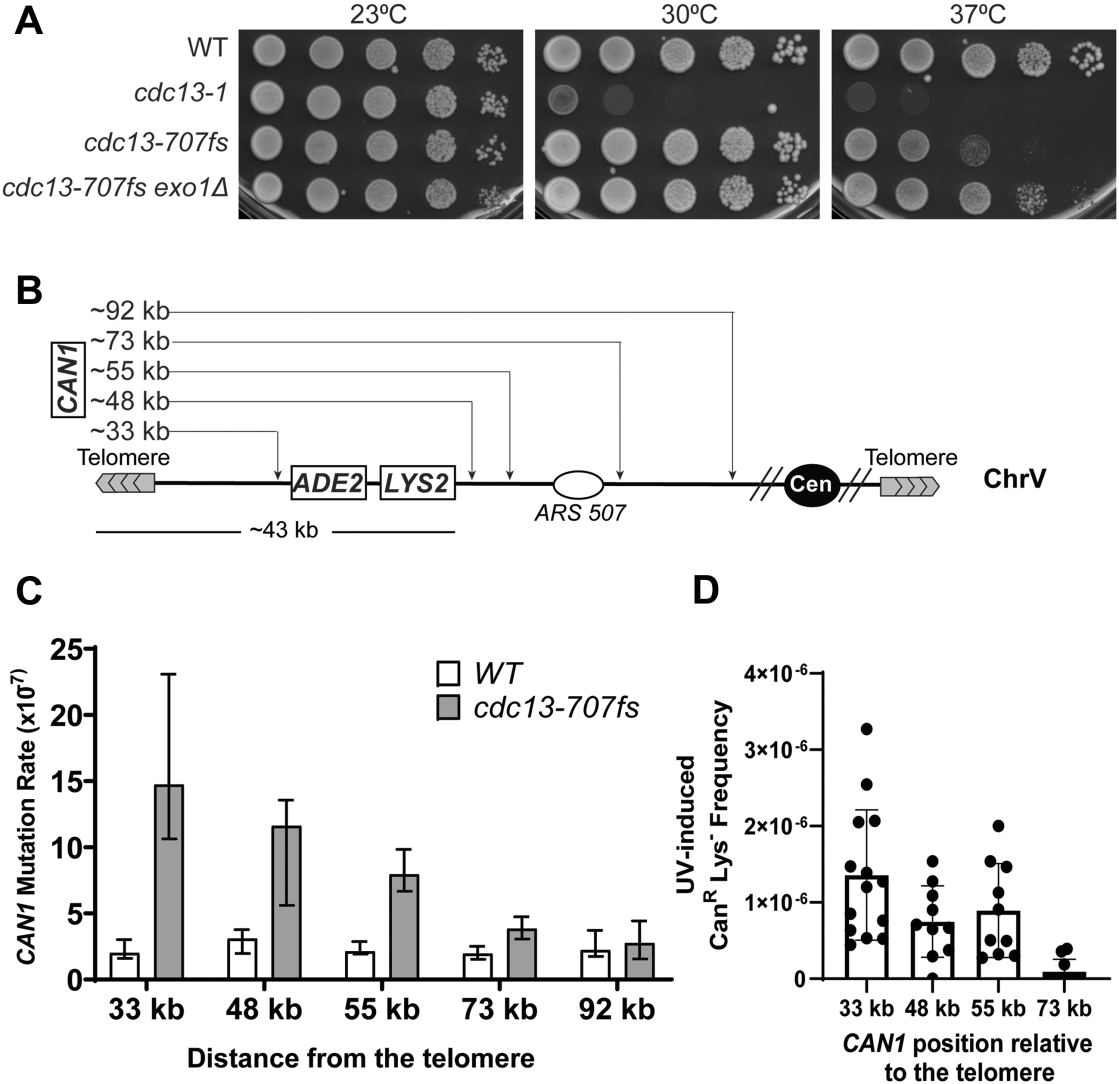
Cell viability and *CAN1* mutagenesis in subtelomeric regions in *cdc13-707fs*. (**A**) Viability of indicated strains at 23, 30 and 37°C in a serial dilution spot test. (**B**) Diagram of *CAN1* locations relative to the telomere on the left arm of chromosome V. (**C**) Mutation rates in *CAN1* in WT and *cdc13-707fs* strains. Data are represented as the median value ± 95% confidence interval. (**D**) Frequency of UV-induced double mutations (Can^R^Lys^−^) in the *cdc13-fs* strain. Canavanine resistant clones from UV-treated (45 J/m^2^) or untreated cells in WT and *cdc13-707fs* strains were assessed for their lysine auxotrophy to determine the frequency of Can^R^Lys^−^ clones. Since no spontaneous double mutations in WT and *cdc13-707fs* and no UV-induced double mutations in WT were recovered, only UV-induced double mutations in *cdc13-707fs* are presented. Data are represented as the mean value ± standard deviation.

A defect in telomere capping is associated with 5′-3′ end resection and ssDNA exposure vulnerable to the accumulation of mutations (38). To gauge the extent of ssDNA formation in *cdc13-707fs*, we analyzed mutagenesis at a *CAN1* reporter located at different distances away from the telomere in strains devoid of *Alu*-IRs (Figure 1B) because the levels of spontaneous mutations in ssDNA are drastically higher than in dsDNA (63–65). We found that at 30°C the mutation rates in *cdc13-707fs* are elevated when *CAN1* is located at 33 kb (∼7-fold), 48 kb (∼4-fold), 55 kb (∼4-fold) and 73 kb (∼2-fold) but not at 92 kb (Figure 1C) away from the telomere. Importantly, the increase in mutagenesis is dependent on Rev3 (Supplementary Table S1), indicating that mutagenesis is a result of restoration synthesis of the resected strand involving Polζ. To confirm that long ssDNA molecules are generated in *cdc13-707fs* strains, we UV-irradiated (45 J/m^2^) strains with *CAN1* located 33, 48, 55 and 73 kb from the telomere and *LYS2* at ∼43 kb. UV irradiation, and DNA damaging agents in general, is a powerful tool to highlight ssDNA since it generates clustered and strand-coordinated mutations otherwise undetectable in dsDNA-containing reporters (38,63–69). We thus measured the frequency of double mutations by scoring for Can^R^ and Lys^−^ colonies. In absence of UV treatment, no double mutations were recovered in WT strains or *cdc13-707fs*. Upon UV treatment, Can^R^Lys^−^ double mutants were recovered only in the *cdc13-707fs* strain at all tested distances except at 92 kb, with the lowest frequency observed at 73 kb (Figure 1D). The occurrence of double mutations cannot be a result of gross chromosomal rearrangements (GCRs) since Can^R^Lys^−^ colonies are Ade^+^ and *CAN1* is located upstream of the essential gene *PCM1* (except at 33 kb), which circumvents chromosomal arm loss (Figure 1B). Moreover, sequencing of double Can^R^Lys^−^ mutants from a strain with *CAN1* located at 33 kb and *LYS2* at 43 kb away from the telomere (Supplementary Figure S2) revealed the presence of coordinated clustered mutations (all double mutants contained 2 to 4 mutations and all 58 identified mutations resulted from mutations in pyrimidine bases located in the unresected strand) (Supplementary Table S2 and Supplementary Figure S2). Altogether, our analysis indicates that in *cdc13-707fs*, ssDNA stretches are very long and can efficiently reach up to 73 kb.

Telomere uncapping using *cdc13-707fs* can be used as a powerful system to conditionally create extensively resected chromosomal DNA, in conditions where high temperatures are not required and viability is not impaired.

### Widely-separated and divergent IRs induce high levels of GCRs in *cdc13-707fs*

Previously, we demonstrated that both the distance between *Alu*-IRs and the level of sequence homology greatly impacts an IR’s ability to stimulate homologous recombination in dsDNA (34). This effect results from a decreased probability of cruciform formation and, hence, a decreased probability of DSBs. Since *cdc13-707fs* scored for a high level of *Alu*-IR fragility when a 94% homologous IR was located ∼43 kb away from the telomere and this region falls into the resection window in the mutant, we addressed the contribution of ssDNA formation to the instability of seemingly stable IRs.

**Figure 2.**
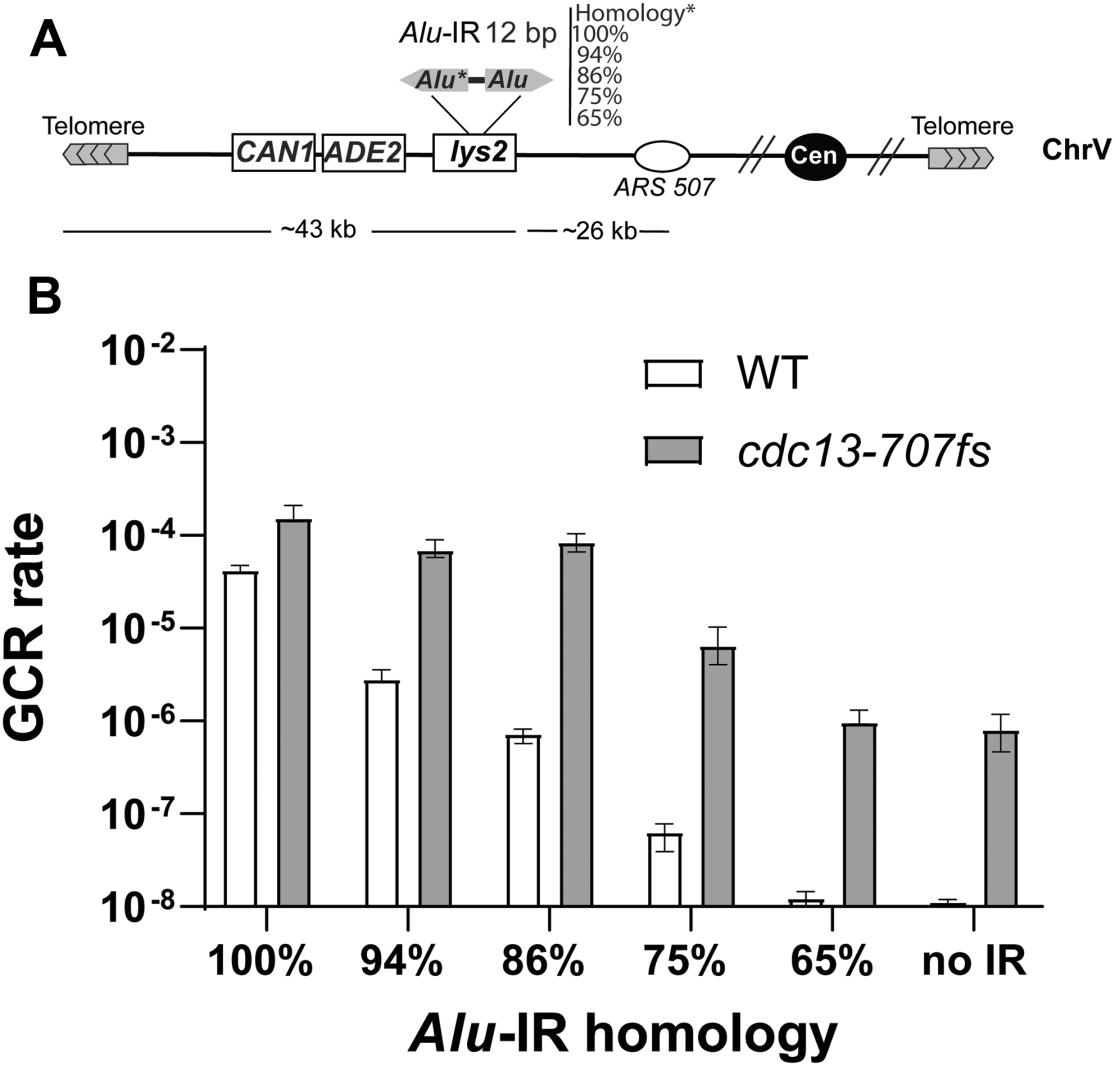
Homeologous IRs induce high GCR levels upon telomere uncapping in *cdc13-707fs*. (**A**) Diagram of the genetic assay to score for GCRs induced by *Alu*-IRs separated by 12 bp and harboring different levels of homology. One of the *Alu*s composing the IR is either identical to the second inverted *Alu* (100%) or exhibits nucleotide changes decreasing the level of homology to 94%, 86%, 75% or 65%. The indicated IRs have been inserted into *LYS2* and are located ∼43 kb away from the left telomere of ChrV. Loss of this region results in GCRs that are manifested as canavanine-resistant Ade^−^ red colonies. (**B**) GCR rates in WT and *cdc13-707fs* strains harboring the indicated divergent *Alu*-IRs with 12 bp spacers. Data are represented as the median value ± 95% confidence interval.

We generated a set of *Alu*-IRs with 100, 94, 86, 75 and 65% of homology (Figure 2A). *Alu*-IRs that are not 100% identical are referred to as divergent IRs. The different *Alu*-IRs were inserted into *LYS2* in strains containing the reporter system for GCRs (Figure 2A). As anticipated for the WT strain, a decrease in the degree of homology from 100% to 94, 86 or 75% decreases the GCR rates by a factor of approximately 15, 58 and 1087, respectively (Figure 2B). At 65% homology, the rate of GCRs reaches the spontaneous level (i.e. without an *Alu*-IR). Interestingly, *cdc13-707fs* shows a stronger induction of GCRs compared to WT at all *Alu*-IRs exhibiting >65% of homology (3.6-fold at 100%, 25-fold at 94%, 116-fold at 86% and 165-fold at 75%). Importantly, and in contrast to WT, decreasing the level of homology of the *Alu*-IRs does not drastically impact their ability to induce GCRs. Indeed, compared to the 100% homologous *Alu*-IR, the fold decrease is only ≤2 at 94 and 86% and 23 at 75% in *cdc13-707fs*. Similar to WT, inverted *Alu*s sharing 65% of homology did not induce a significant increase in GCRs compared to the strain without an IR.

**Figure 3.**
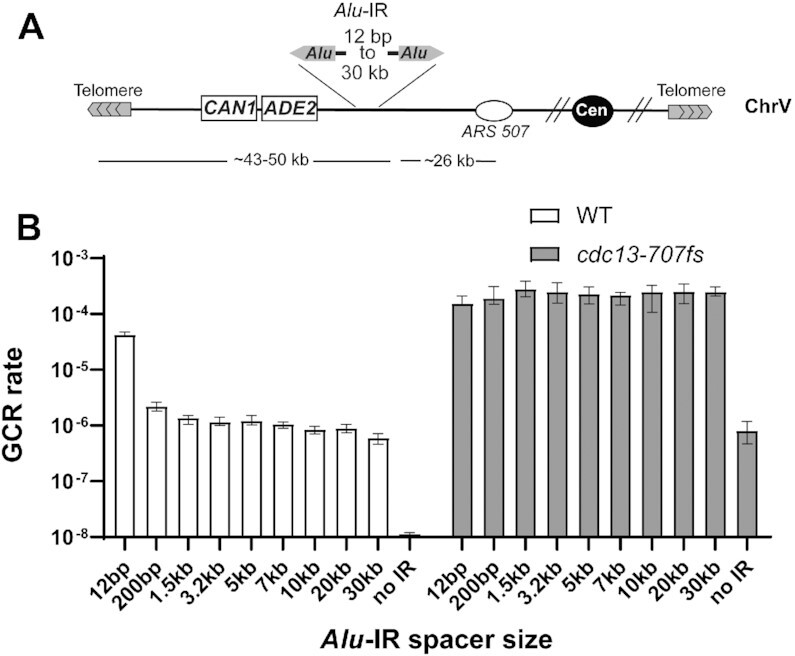
Distantly separated inverted repeats induce high GCR levels upon telomere uncapping in *cdc13-707fs*. (**A**) Diagram of the genetic assay to score for GCRs induced by *Alu*-IRs separated by asymmetrical spacers ranging from 12 bp to 30 kb. *Alu*-IRs separated by 12, 200, 1.5, 3.2, 5 and 7 kb were inserted into *LYS2*. The resulting IRs are located ∼43 to 50 kb away from the telomere. In order the keep the *Alu*-IRs with 10, 20 and 30 kb spacers <50 kb away from the telomere, another strategy was used to generate the strains harboring them (see Material and methods section). (**B**) GCR rates in WT and *cdc13-707fs* strains harboring *Alu*-IRs with the indicated spacer. Data are represented as the median value ± 95% confidence interval.

In addition to the degree of homology, the distance between IRs is an important factor dictating its fragility potential. For example, increasing the spacer size from 12 bp to 100 bp decreases IR-induced recombination by 300-fold in dsDNA (34). Here, we compared the effect of increasing distances in the spacer size of *Alu*-IRs on promoting GCRs in WT and *cdc13-707fs*. For this, we generated a set of *Alu*-IRs separated by up to 30 kb (Figure 3A). The strains harboring these IRs are built in such way that the centromere-proximal *Alu* is located <50 kb away from the telomere (see Material and methods), and thus still in the resection window in *cdc13-707fs*. In WT, increasing the spacer size from 12 bp to 200 bp led to a ∼20-fold reduction in GCR rates (Figure 3B). Increasing spacer size to 1.5 or 20 kb reduced GCR rates by a factor of 31 and 47, relative to the 12 bp spacer. The *Alu*-IR with 30 kb spacer strain showed the lowest GCR rate (71-fold decrease) but remained higher than the spontaneous level (Figure 3B). These results are in agreement with the fact that decreasing the IR spacer size provides more opportunity to form secondary structures and indicate that IRs separated by long distances (up to 30 kb) may occasionally interact. In stark contrast to WT, the *cdc13-707fs* mutant showed the same GCR rates regardless of the spacer size (Figure 3B).

These data show that the interaction between two inverted sequences is not only highly enhanced in *cdc13-707fs* upon telomere uncapping and DNA resection but tolerates a high level of divergence (up to 25%) and large spacers (up to 30 kb) to lead to chromosomal rearrangements. This implies that relatively stable IRs that were not considered to be a threat to genome stability acquire the potential to destabilize the genome when encompassed in regions of ssDNA. Consistent with this, deletion of *EXO1* in *cdc13-707fs* suppresses the increase of IR-induced GCRs (Supplementary Table S3).

### Interaction of IRs in ssDNA leads to genome instability through the formation of a dicentric chromosome

**Figure 4.**
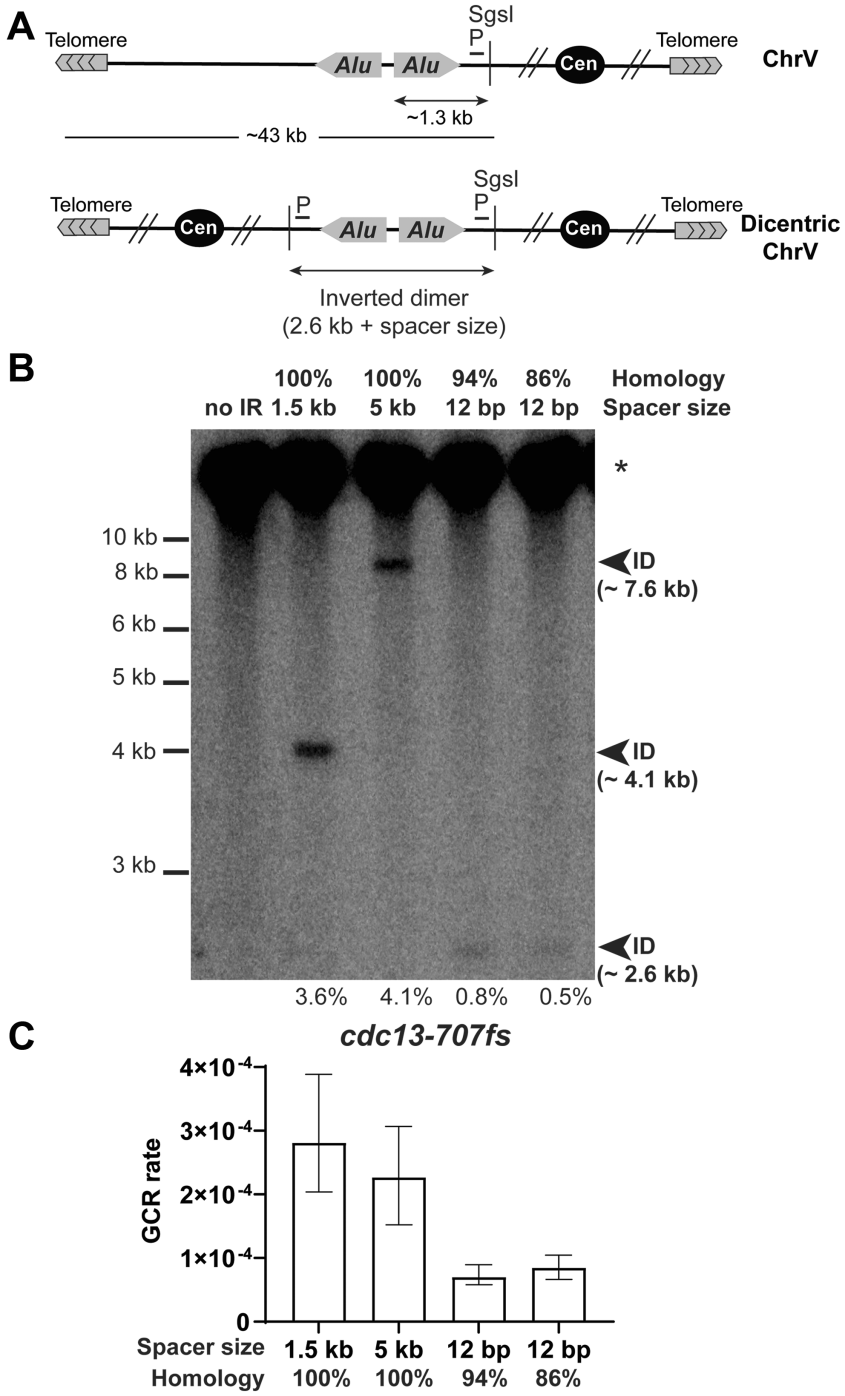
Inverted dimer formation in distantly-spaced and homeologous IRs upon telomere uncapping. (**A**) Scheme of chromosome V and inverted dicentric chromosome V. The sizes of the DNA fragments after SgsI digestion and the location of the centromere-proximal probe (P) used are indicated. (**B**) Inverted dimer detection in *cdc13-707fs* strains containing the indicated *Alu*-IR. Genomic DNA was embedded into agarose plugs and digested with SgsI. The 43–48 kb fragment corresponding to the unchanged chromosome V (*) and inverted dimers (ID) were highlighted using the centromere-proximal probe. The expected size of the ID for the different *Alu*-IRs is indicated in brackets. The percentage of dimer relative to the intact ChrV is indicated below. **(C**) GCR rates in *cdc13-707fs* strains harboring the indicated *Alu*-IRs. Data are represented as the median value ± 95% confidence interval.

Our data indicate that structural parameters that normally constrain the instability of IRs are overcome when the repeats are interacting in *cis* in the context of ssDNA. This prompts us to propose that GCRs in *cdc13-707fs* are not induced because of an increase in DSBs, but rather they originate from a DNA fold-back mediated by the annealing of the ssDNA IR followed by dicentric chromosome formation, its breakage in anaphase and repair events. To test this hypothesis, we analyzed inverted dimer (Figure 4A) formation in WT and *cdc13-707fs* strains containing *Alu*-IRs separated by 1.5 kb. No bands corresponding to DSBs were detected in any strains (Supplementary Figure S3). However, we observed inverted dimer formation in *cdc13-707fs* strains with the 1.5 kb spacer *Alu*-IRs (Figure 4B and Supplementary Figure S3). Similarly, we found that *Alu*-IRs separated by a 5 kb spacer, as well as *Alu*-IRs harboring 94% and 86% of homology, also gave rise to inverted dimer formation in *cdc13-707fs*. Detection of inverted dimers shows that dicentric chromosomes accumulate in the population of *cdc13-707fs* cells propagated at the semi-permissive temperature. Therefore, the mechanism by which divergent and distantly separated IRs induce chromosome fragility is through dicentric chromosome formation, breakage of which leads to gross chromosomal rearrangements. We noticed that GCR rates in *cdc13-707fs* are higher in the strains with *Alu*-IRs with long spacers compared to the strains with divergent *Alu*-IRs (Figure 4B). This suggests that IRs separated by long spacers are more permissive to annealing than shortly spaced but divergent IRs when contained in ssDNA. Also, sequence divergence is a stronger barrier in ssDNA for IR interaction than a separation by a long distance.

### Genetic dependency of IR-mediated dicentric chromosome formation in *cdc13-707fs*

The results above show that chromosome fragility induced by divergent and distantly separated IRs in *cdc13-707fs* is independent of DSB formation and likely relies on two events prior to dicentric chromosome formation: (i) annealing of the two inverted *Alu* sequences and (ii) removal of the non-annealed telomere-proximal tail generated by the fold-back. Therefore, we tested the genetic dependency of IR-mediated inverted dimer formation in *cdc13-707fs* by targeting factors involved in single-strand DNA annealing (SSA), like Rad52-Rad59, and the resolution of 3′-flap structures.

**Figure 5.**
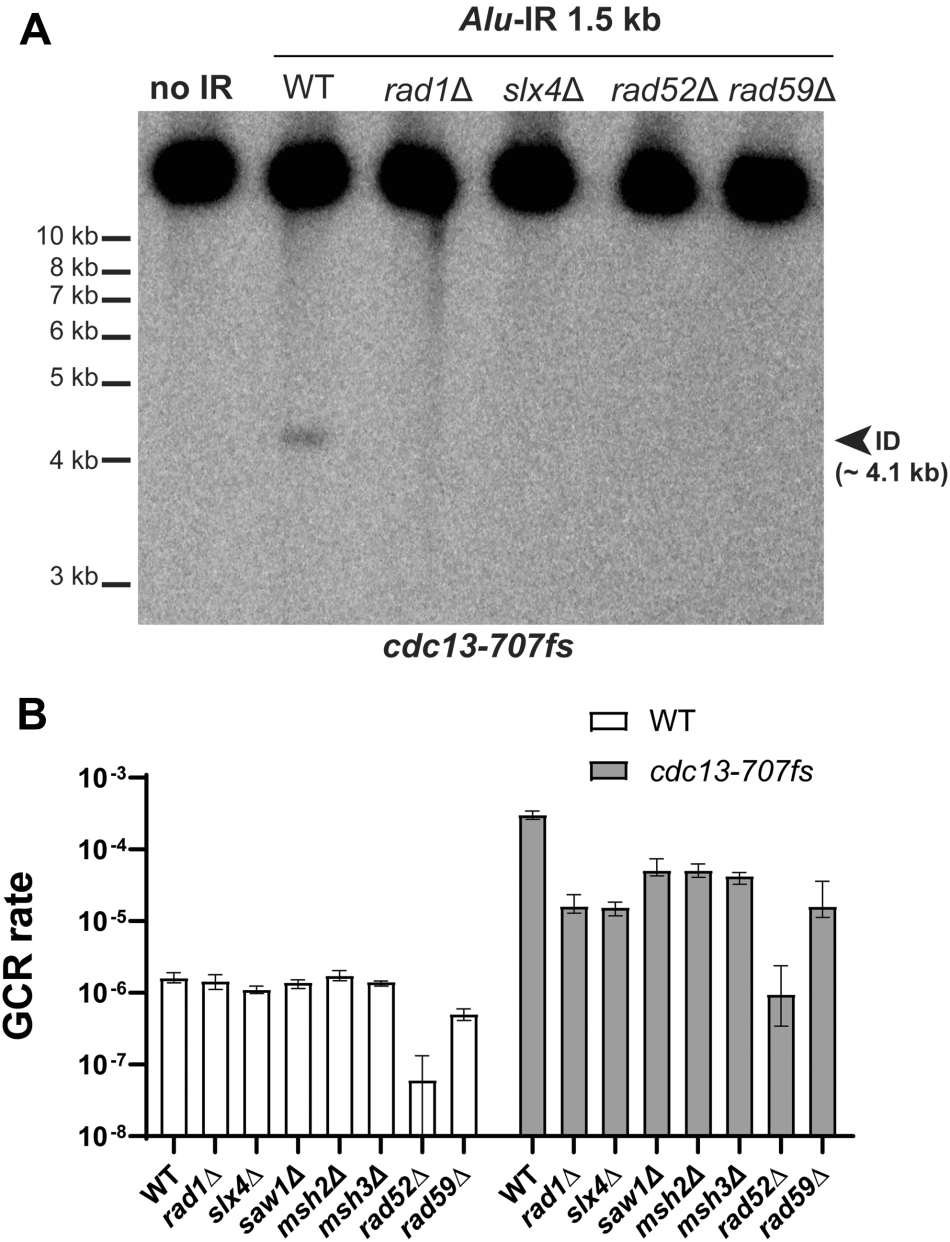
Genetic dependency of inverted dimer formation upon telomere uncapping. (**A**) Inverted dimer detection in *cdc13-707fs* strains containing *Alu*-IRs with a 1.5 kb spacer and deleted for *RAD1*, *SLX4*, *RAD52* or *RAD59*. Detection was performed as described in Figure 4. (**B**) GCR rates in the indicated strains harboring an *Alu*-IR with a 1.5 kb spacer. Data are represented as the median value ± 95% confidence intervals.

Strains deleted for either *RAD52* or *RAD59* in the *cdc13-707fs* background did not show a signal corresponding to the inverted dimer formed by the 1.5 kb spaced *Alu*-IR (Figure 5A). This suggests that intra-stand annealing between IRs in ssDNA is mediated by Rad52 and Rad59. Consistent with this, the GCR rates were decreased in both *rad52*Δ and *rad59*Δ, with the deletion of *RAD52* showing the strongest decrease (∼300-fold). The dramatic effect of *RAD52* deletion on *Alu*-IR-induced GCRs can be explained by the fact that, in addition to mediating ssDNA annealing, Rad52 is required for DNA repair and the recovery of GCR events (70,71). Accordingly and expectedly, deletion of *RAD52* in strains with the 1.5 kb spaced *Alu*-IR and functional Cdc13 reduced the GCR rate by ∼23-fold (Figure 5B).

In the absence of Rad1 (endonuclease of the Rad1-Rad10-Slx4 complex), no signal corresponding to the inverted dimer was detected in *cdc13-707fs* strains harboring the 1.5 kb spacer *Alu*-IR (Figure 5A). In agreement with this, deletion of *RAD1* led to a 19-fold reduction in GCR rates in *cdc13-707fs* whereas in WT the GCR rates remained unaffected. These data suggest that, following IR interaction in ssDNA, the dicentric inverted chromosome is formed less efficiently in the absence of Rad1-Rad10. We also tested the effect of several factors involved in the Rad1-Rad10-dependent removal of 3′ nonhomologous tails such as Slx4, Saw1 and Msh2-Msh3 (72). Similar to the effect of *RAD1* deletion, deletion of *SLX4* in *cdc13-707fs* reduced the *Alu*-IR-induced GCRs by 19-fold and the inverted dimer was undetectable in the double mutant (Figure 5A and B). *Alu*-IR-induced GCRs were also decreased upon deletion of *SAW1*, *MSH2* and *MSH3* (Figure 5B) but to a lesser extent than *RAD1* and *SLX4* (6-fold decrease versus 19-fold). In *CDC13*, 1.5 kb spaced *Alu*-IR background, deletion of each of these genes did not impact GCR rates (Figure 5B). These data suggest that, following IR interaction in ssDNA, the dicentric inverted chromosome is formed less efficiently in the absence of Rad1-Rad10 and the factors modulating the Rad1-Rad10-dependent removal of 3′-flaps.

### Structural analysis of GCRs induced by IR interaction in ssDNA

Collectively, these data indicate that GCRs mediated by distantly-spaced *Alu*-IRs in *cdc13-707fs* are the consequence of breakage of dicentric chromosomes during anaphase. In this case, rearrangements are expected to show a specific pattern similar to the rearrangements observed in response to hairpin-capped breaks (22): *Alu*-IR proximal inverted duplication and healing via either telomere addition or break-induced replication (BIR) involving a breakpoint near repetitive elements like *Ty* or *delta* elements. BIR-mediated healing results in a translocation event involving a non-homologous chromosome. We analyzed the nature of the rearrangements of 12 Can^R^Ade^−^ clones derived from *cdc13-707fs* harboring the 1.5 kb spaced *Alu*-IR by whole genome sequencing. All clones exhibit a terminal deletion and an inverted duplication of chromosome V, reflecting dicentric chromosome breakage. The duplications span over ∼24 kb (in 8 out 12 clones) or ∼100 kb (in 4 out 12 clones), with a breakpoint located near the *PAU2* gene (generating ∼24 kb duplication) or *Ty1* element or lysine tRNA gene (generating ∼100 kb duplication) (Supplementary Table S4 and Supplementary Figure S4). None of the analyzed clones showed telomeric addition but rather a translocation event often involving one of the subtelomeric *PAU* genes or *Ty1* elements located on different chromosomes (Supplementary Table S4). These data are consistent with a proposed mechanism of GCRs in *cdc13-707fs* mutants via the interaction of IRs in ssDNA, inverted dimer formation, breakage in anaphase and repair. The signatures of these GCRs are similar to the ones generated by hairpin-capped breaks at *Alu*-QPs (22). However, the repair events following anaphase breakage are different and might reflect the status of the subtelomeric chromatin in wild-type and *cdc13-707fs* mutants (see Discussion below).

### Analysis of *Alu*-IRs distribution in regions of ssDNA in the human genome

With over one million copies, the *Alu* element is the highest copy number transposon in the human genome (73,74). Besides their insertional capabilities, *Alu* elements are the most important reservoir of homologous/homoeologous sequences in the human genome and thus a potent source for genome rearrangements (75). Distantly spaced *Alu* pairs are found both in direct and inverted orientations (76). It has been established that long ssDNA can form accidentally in the genome during several DNA transactions, especially when DNA damage and breakage are involved (38). In cancer genomes, these regions are a perfect substrate for hypermutation by ssDNA specific APOBEC cytidine deaminase(s) leaving a permanent record of mutation clusters highly enriched with the APOBEC hypermutation signature (77,78). Here, we mapped dangerous *Alu*-IR pairs as well as other transposable element (TE) pairs forming an IR in the human genome in regions that, by the presence of APOBEC hypermutated clusters, experienced formation of long ssDNA stretches. ssDNA regions in the history of 336 APOBEC hypermutated human tumors, including Bladder-transitional cell carcinoma (Bladder-TCC), breast adenocarcinoma (Breast-AdenoCA), Cervix-squamous cell carcinoma (Cervix-SCC), Head-SCC, Lung-AdenoCA and Lung-SCC were analyzed (78). It was found that these regions of hypermutated ssDNA can be long and span up to 150 kb. Based on the parameters for efficiently interacting *Alu*-IRs in ssDNA we identified in yeast, we mapped all TE inverted pairs that are separated by <30 kb and have >75% homology in ssDNA regions in the human genome.

**Figure 6.**
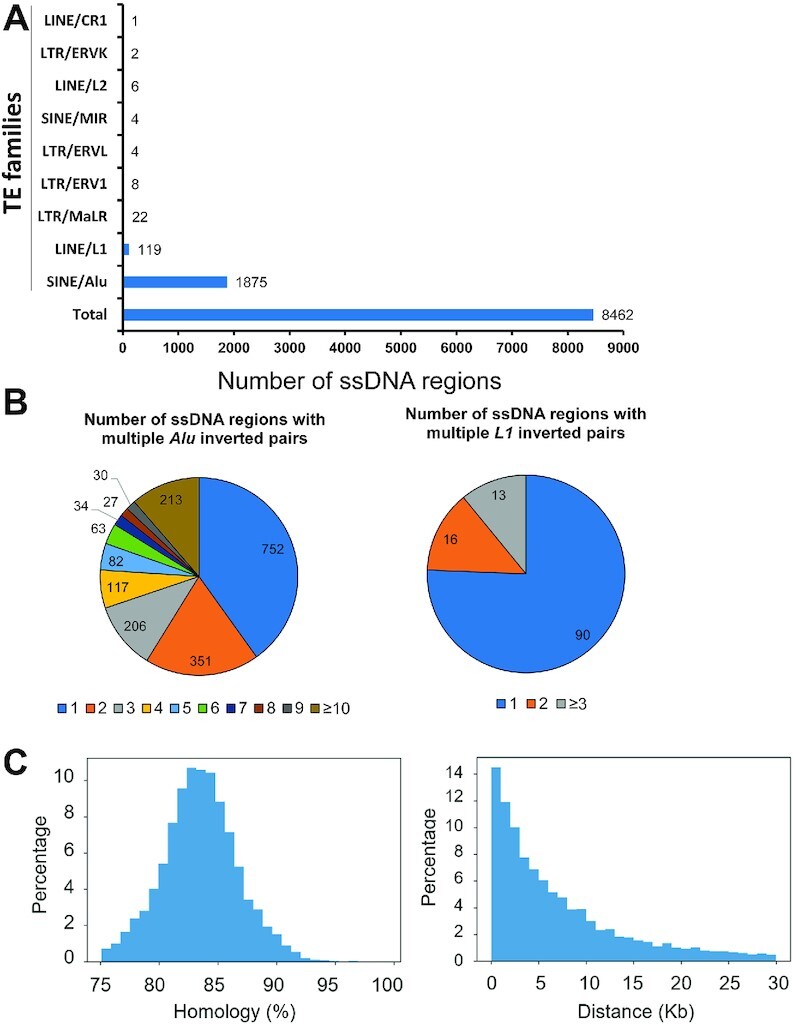
Analysis of IRs within ssDNA regions in human cancers. The presence of IRs composed of transposable element (TE) pairs in an inverted orientation was assessed in the genomic regions showing clustered and strand coordinated base substitutions identified as APOBEC-mutational signatures. The mutation clusters were identified in APOBEC-hypermutated cancer genomes that include bladder, breast, cervix, head and neck and lung cancers (Sakofsky *et al.*, 2019). Each cluster corresponds to ssDNA regions vulnerable to APOBEC-induced mutagenesis (**A**). Numbers of clusters containing an inverted TE pair. The different types of TEs are represented on the *Y*-axis. The threshold for inverted TE pair calling in the clusters was set at <30 kb in terms of distance separating the TEs of each pair and >75% homology between the TEs of each pair. (**B**) Distance distribution in *Alu* and *L1* pairs identified in (A). (**C**) Homology distribution in *Alu* and *L1* pairs identified in (A).

Among the 8462 regions analyzed, 2041 (24%) were found to contain a TE inverted pair (Figure 6A), with SINE/*Alu* being the major TE family represented (22%) followed by LINE/*L1* (1.4%). Among the 1875 regions with *Alu*-IRs, 60% contained more than one inverted *Alu* pair (Figure 6B). Interestingly, 213 regions show the presence of >10 *Alu*-IRs. The identified *Alu* inverted pairs were classified according to their degree of homology and spacing (Figure 6C). The vast majority exhibit between 80 and 90% identity, and most of the inverted *Alu* pairs are separated by <10 kb. This analysis reveals that a substantial number of *Alu*-IRs exhibiting the same features as the ones we showed to be hyper-GCR prone in *cdc13-707fs* can fall in long ssDNA regions formed in the history of the human tumors. The presence of a large number of inverted TEs in ssDNA regions in in APOBEC hypermutated human tumors shows that ssDNA formation offers the opportunity for IRs to interact.

## DISCUSSION

We have investigated structural determinants of the physical interaction in ssDNA between *Alu*-IRs using the *cdc13-707fs* allele. Expression of *cdc13-707fs* at a semi-permissive temperature leads to telomere uncapping and massive DNA resection. ssDNA formation in this mutant can efficiently reach at least 73 kb away from the telomere. Here, we measured ssDNA formation by analyzing the occurrence of strand-coordinated and damage-induced mutations because of the robustness and sensitivity of this tool (63). Previously, it was determined (by ssDNA-qPCR) that ∼15 kb of ssDNA accrue in *cdc13-1* strains (48) whereas the analysis of damage-induced strand-coordinated mutation clusters showed that stretches of ssDNA can reach up to 50 kb (38). By moving the *CAN1* reporter along the left arm of chromosome V, we demonstrated here that the extent of ssDNA formation in CST-deficient yeast cells can be greater than previously assessed and spans somewhere between 73 and 92 kb.

Expression of CST hypomorphic alleles was shown to increase chromosomal fragility at triplex-forming GAA/TTC trinucleotide tracts as well as their expansions (52). Similarly, it was found that GAA/TTC expansions and contractions as well as an increase GCRs, are induced in the *cdc13-1* mutant (53). The genetic reporters used in these studies were located <80 kb away from the telomere. In light of our results, the instability of the DNA repeats observed in CST deficient cells may be due to a ssDNA context that imparts a higher probability of forming secondary structures triggering chromosomal fragility. In accordance with this, GAA expansions in the *cdc13-1* mutant are completely eliminated in the absence of the major nuclease responsible for telomere end resection Exo1.

**Figure 7.**
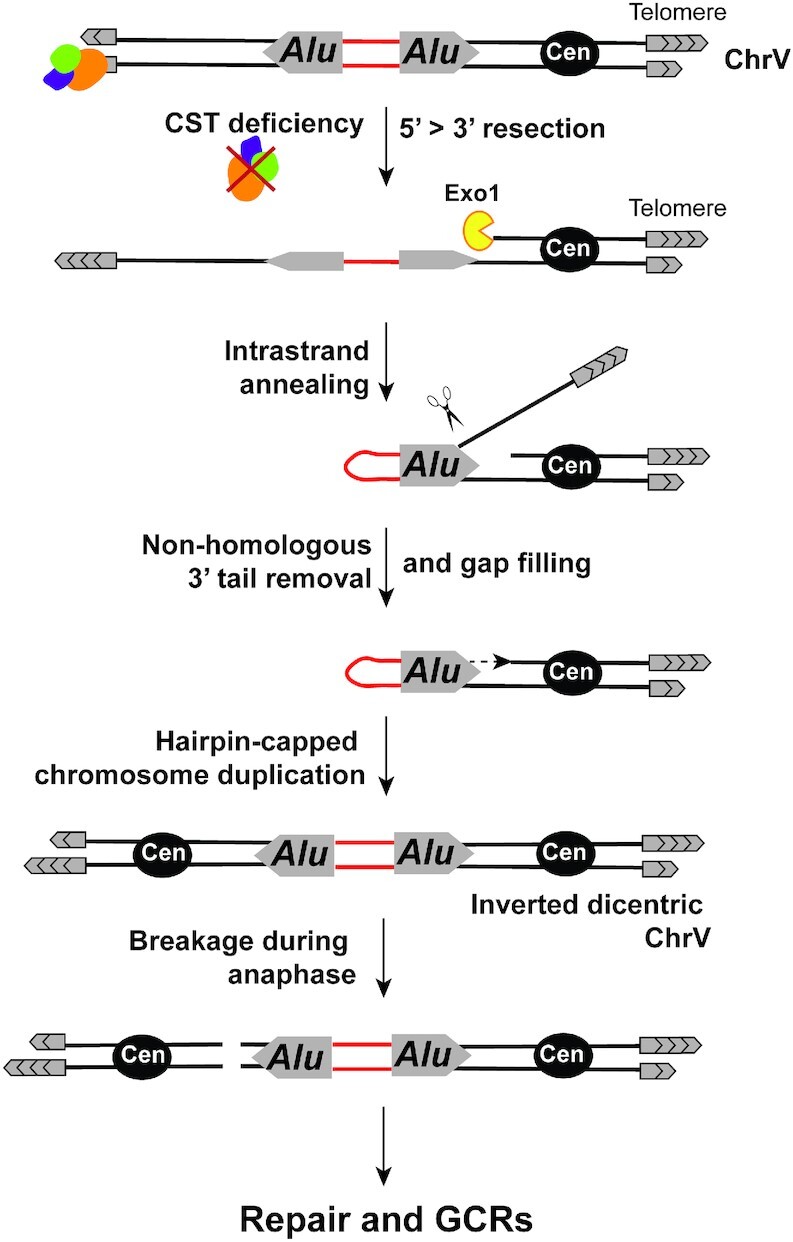
Mechanism of IR-mediated GCR induction as a result of telomere uncapping. See discussion for details.

Using *cdc13-707fs* as a tool to conditionally induce telomere uncapping and generate long stretches of ssDNA, we were able to identify how the interaction between differently-spaced and divergent IRs promotes GCRs and propose a model for IR-mediated GCR induction as a result of telomere uncapping (Figure 7). CST deficiency leads to C-strand resection generating long (up to 73 kb) stretches of ssDNA. Although ssDNA is only formed in a subset of cells upon *cdc13-707fs* expression at the semi-permissive temperature, the GCR assay is sensitive tool to reveal the outcome of IR interactions in ssDNA. Homologous and divergent IRs can efficiently anneal even when they are separated by a long distance (up to 30 kb). A fold-back generates a hairpin with an unannealed 3′-ending flap resolved by the Rad1-Rad10-Slx4 complex in coordination with Msh2-Msh3 and Saw1. DNA synthesis proceeding toward the centromere results in a hairpin-closed chromosome, duplication of which leads to dicentric chromosome formation. During anaphase, the dicentric chromosome breaks asymmetrically, resulting in a duplication of the sequences adjacent to the deletion. Our sequencing analysis of the resulting rearrangements shows that the broken chromosomes acquire telomeres exclusively via an invasion into a nonhomologous chromosome during BIR. This highlights two noticeable differences compared to hairpin-capped break-mediated GCRs observed in WT cells. First, no telomere addition was detected in contrast to the GCR pattern observed in response to hairpin-capped break formation (22). This is consistent with the involvement of Cdc13 in *de novo* telomere addition at DSBs (79,80). The second difference relates to the sequence of the nonhomologous chromosome used as a template in BIR. While chromosome translocations involve mostly a *Ty1*/*delta* element during hairpin-capped break-mediated GCR formation in WT, *Alu*-IR-mediated GCRs in *cdc13-707fs* preferentially show (67%) a translocation breakpoint near a *PAU* gene. Belonging to the largest gene family in *S. cerevisiae*, *PAU* genes are duplicated genes that share high sequence homology (81). Among the 24 existing *PAU* genes, 6 of them were found at a translocation breakpoint in the analyzed GCR events in *cdc13-707fs*. The common features of the *PAU* genes (*PAU4, 9, 12, 13, 18* and *20*) detected at the translocation breakpoint are: (i) they show an opposite polarity compared to the original *PAU2* gene (*Alu*-IR proximal) and thus the same polarity with the duplicated *PAU2* and (ii) they are located in sub-telomeric regions. Indeed, the remaining 18 *PAU* genes not involved in translocation events either have the same polarity as the original *PAU2* gene or are not sub-telomeric. The fact that chromosome healing in the course of GCR formation occurs preferentially via invasion of sub-telomeric regions in *cdc13-707fs* suggests that the sub-telomeric regions become permissive repair substrates upon telomere uncapping, conceivably by changing the chromatin landscape. Therefore, simultaneous uncapping of several chromosomal ends is a possible explanation for the involvement of subtelomeric *PAU* genes during GCR formation.

CST complex defects lead to an increase in GCRs even in the absence of fragile motifs as observed in *cdc13-707fs* (Figure 2B), *cdc13-1* and *cdc13-F684S* (45,53). Spivakovsky-Gonzalez *et al.* proposed that GCR formation is enhanced in CST deficient cells because of a more efficient telomere addition due to the telomere-capping deficiency and telomere elongation. However, this assumption is challenged by the fact that Cdc13 is required for *de novo* telomere addition at DSBs (80). Langston *et al.* suggested that ssDNA formation is the initial event leading to chromosome instability in Cdc13 defective cells and speculate that the unstable chromosomes arise from a one-ended double-strand break. In light of the results of this study, another possible explanation for the induction of GCRs in CST-deficient cells is the interaction between short inverted repeats naturally present in the resected chromosomes V. In agreement with this, Deng *et al.* proposed a mechanism whereby during ssDNA exposure intra-strand annealing between short inverted repeats occurs and forms a foldback structure leading to GCRs (82). The involvement of short inverted repeats in inverted chromosome duplications was also reported upon telomere losses (in telomerase deficient mutants) (83).

We noted that in WT cells, IRs separated by ≥1.5 kb and up to 30 kb induce similar levels of instability that are significantly higher than the background level (strain without IRs). Genome destabilization potential of spaced IRs (1–5.5 kb spacer) was reported in several studies (32,33,35). Two studies in budding and fission yeast indicate that a replication-based mechanism leading to the fusion of spaced IRs and dicentric formation might underly GCR formation (32,33).

A number of studies described the occurrence of a DNA foldback at inverted repeats when DSBs occur nearby (39,44,82,84–86). Here, we systematically investigated the limits of IR interaction in ssDNA outside of the context of DSB formation. Importantly, increasing the distance between IRs to 30 kb does not impact their *cis*-interaction in ssDNA, indicating that IRs might efficiently anneal even at longer distances. The length of the spacer limiting IR interaction and fold-back remains to be determined. The level of homology has a stronger impact on IR interaction compared to the spacer length. However, it is only when the level of homology is decreased <75% that the GCR potential of IRs is lessened in ssDNA. Since the human genome is riddled with *Alu* sequences exhibiting the structural parameters allowing their *cis*-interaction, we propose that the spatial relationship of repetitive elements, inverted *Alu* elements in particular, and thus their probability of acting as rearrangement substrates should be looked at with a new perspective. Importantly, we found that long ssDNA occurring in the history of cancer cells contained inverted TEs with the potential to interact. Whether their *cis*-interaction in this context leads to rearrangements such as terminal deletions and inverted duplications mediated by dicentric formation has to be investigated. It is worth noting that several terminal deletions accompanied by inverted duplications are associated with genetic diseases in humans (e.g. (87–89)). A fold-back mechanism instigated by a DSB and producing a dicentric inverted repeat has been proposed to be at the origin of large inverted duplications (12,84).

In humans, the CST complex is required to maintain telomere integrity and also has essential roles in replication and DNA repair. More recently, a DSB end protection role, limiting resection, has been attributed to the CST complex (90,91). Depletion of Ctc1, the human homolog of Cdc13, triggers the DNA damage response and promotes formation of chromatin bridges (92,93). Based on our results, we propose that the mitotic defects observed in CST-deficient cells might emanate from dicentric chromosomes that form following ssDNA-mediated interaction between divergent IRs. The source of ssDNA may originate at the telomeres, from different regions of the genome undergoing resection, or replicative dysfunction. Recent studies provided evidence that ssDNA gap accumulation impacts genome integrity and response to chemotherapy. Several studies pointed out that BRCA-deficient cancer cells, as well as CST-deficient cells, suffer from an excess of toxic replicative ssDNA gaps (94–97). Our data suggest that the interaction between distantly spaced and divergent inverted repeats can contribute to ssDNA toxicity.

## DATA AVAILABILITY

The published article includes all datasets generated or analyzed during this study. Nanopore sequencing data are available at http://www.ncbi.nlm.nih.gov/bioproject/899891.

## Supplementary Material

gkad153_Supplemental_Files
