# Supplementary material for: Widely spaced and divergent inverted repeats become a potent source of chromosomal rearrangements in long single-stranded DNA regions
Source: Nucleic Acids Res. 2023 Mar 15;51(8):3722–34. doi: 10.1093/nar/gkad153 (PMC10164571; doi:10.1093/nar/gkad153)
Supplement: gkad153_Supplemental_Files [file gkad153_supplemental_files.zip › Ait Saada et al 2022 Supplemental data revised.pdf]

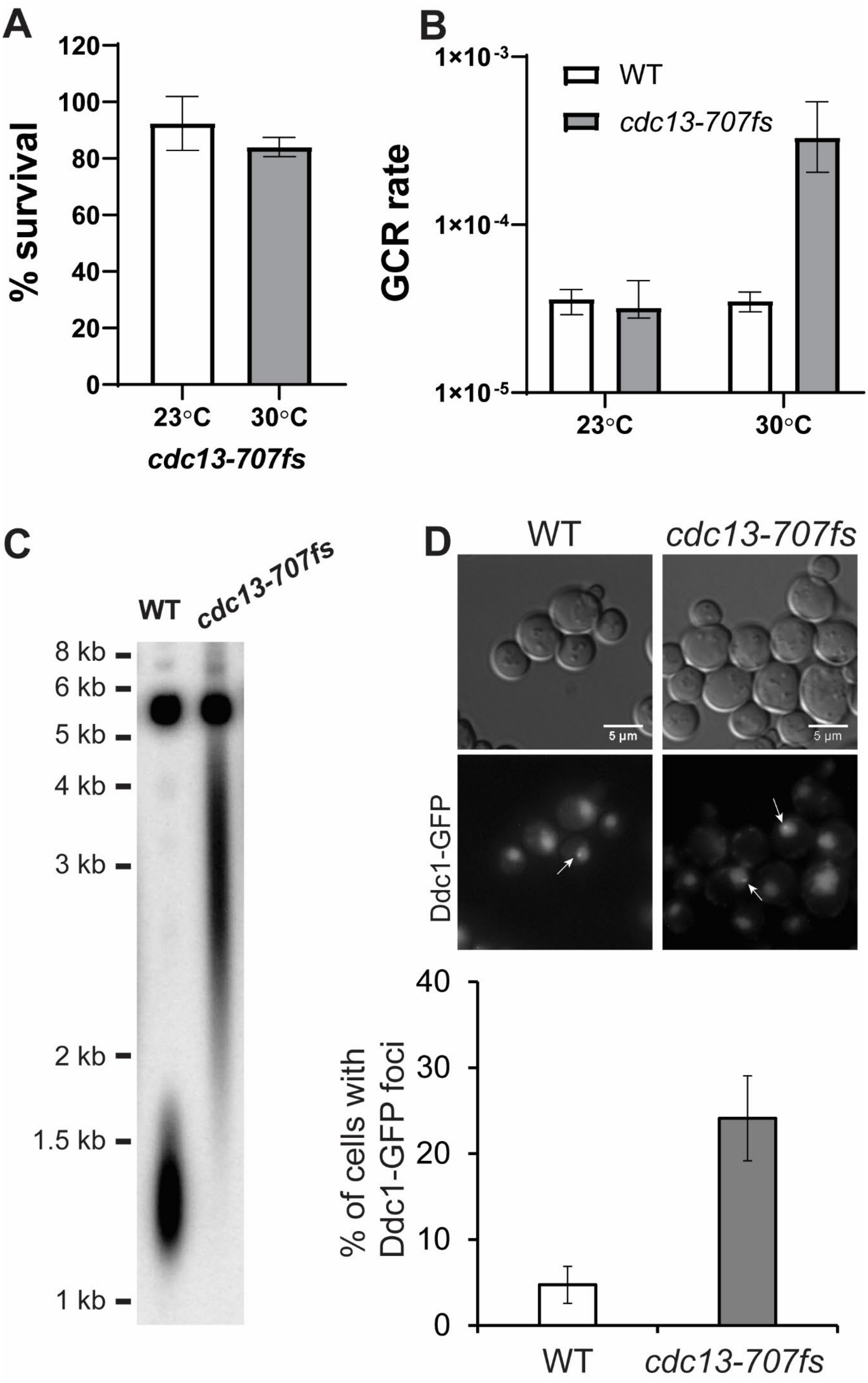

A
120
100
80
60
40
20
0
% survival
23°C
30°C
cdc13-707fs
B
1×10-3
1×10-4
1×10-5
GCR rate
WT
cdc13-707fs
23°C
30°C
C
WT
cdc13-707fs
8 kb
6 kb
5 kb
4 kb
3 kb
2 kb
1.5 kb
1 kb
D
WT
cdc13-707fs
5 µm
5 µm
Ddc1-GFP
40
30
20
10
0
% of cells with Ddc1-GFP foci
WT
cdc13-707fs

**Figure S1. *cdc13-707fs* growth at 30 °C does not impair cells viability but impacts telomere length and *Alu*-IR-mediated GCRs. Related to Figure 1.**

**A.** Cell viability of *cdc13-707fs* strains grown at 23 or 30 °C. Data are represented as the average value ± standard error of the mean. **B.** GCR rates in WT and *cdc13-707fs* strains harboring the 12 bp spacer *Alu*-IR and grown at the indicated temperatures. Data are represented as the median value ± 95% confidence interval. **C.** Telomere length analysis upon telomere uncapping in *cdc13-707fs*. Genomic DNA was extracted and digested with XhoI. After separation in a 1% gel, telomeric fragments were highlighted with Y'-specific probe. **D.** Ddc1-GFP imaging in WT and *cdc13-707fs* cells grown at 30 °C. **Top panel:** Example of cells showing Ddc1-GFP foci (white arrows). **Bottom panel:** quantification of the percentage cells showing Ddc1-GFP. Data are represented as the average ± 95% confidence interval.

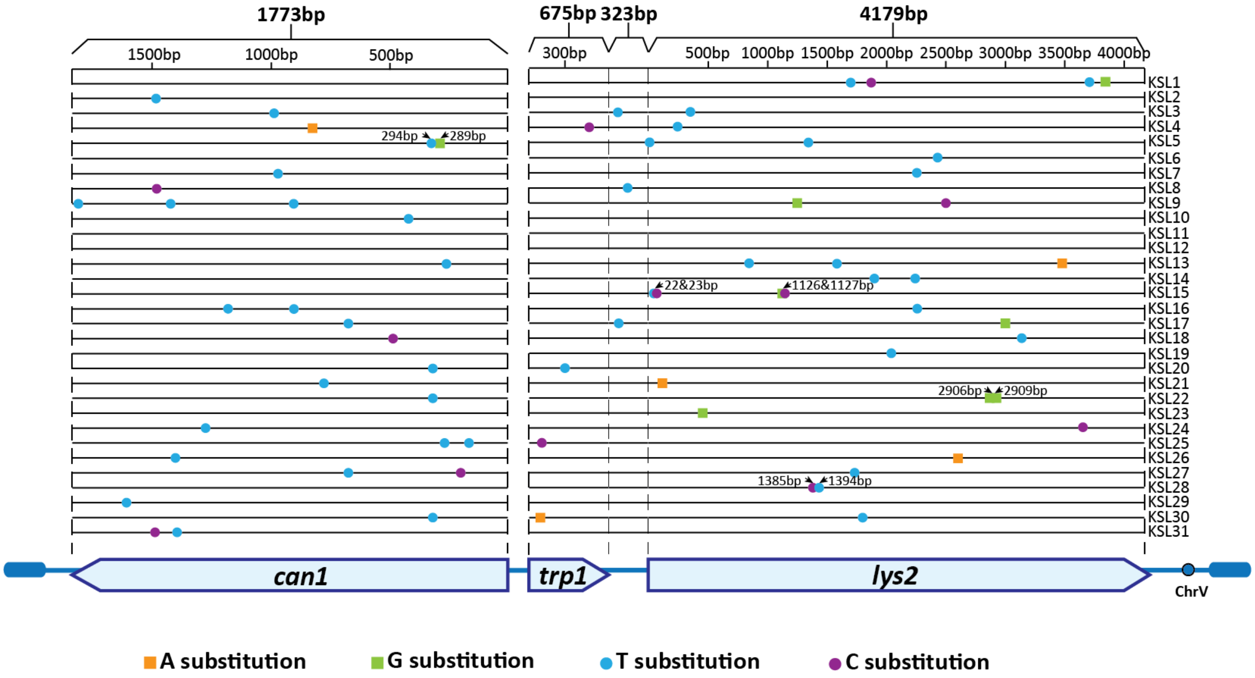


**Figure S2. Simple base substitutions from 31 isolates on *CAN1TRP1LYS2* cassette after 45 J/m2 UV irradiation. Related to Figure 1.**

31 $Can^R Lys^-$ isolates are represented. Single nucleotide changes at A (orange), G (green), T (blue), and C (purple) in the unresected strand are shown in *CAN1*, *TRP1* and *LYS2*. Gene sizes are indicated on top. Indels are not shown.

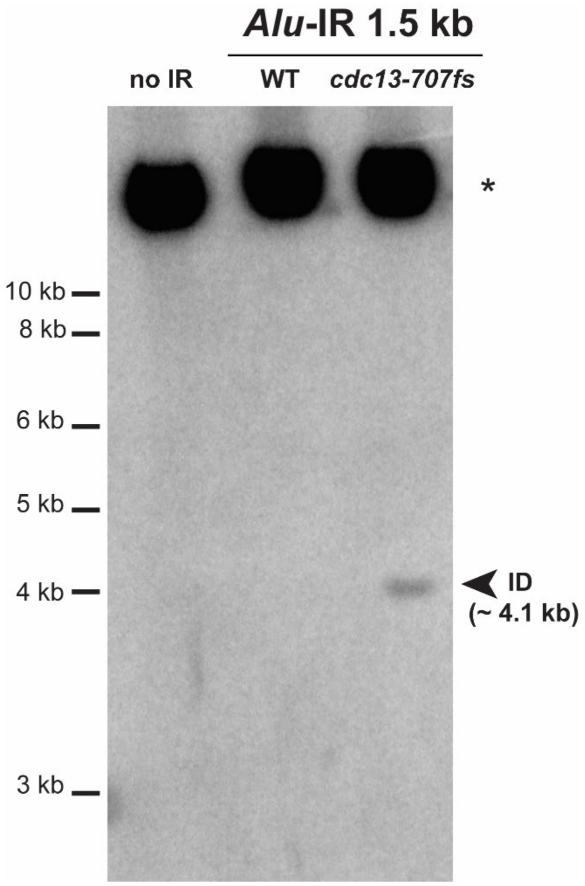


**Figure S3. Inverted dimer formation in *Alu*-IRs separated by 1.5 kb upon telomere uncapping. Related to Figure 4.**

Detection was performed in the indicated strains as described in Figure 4.

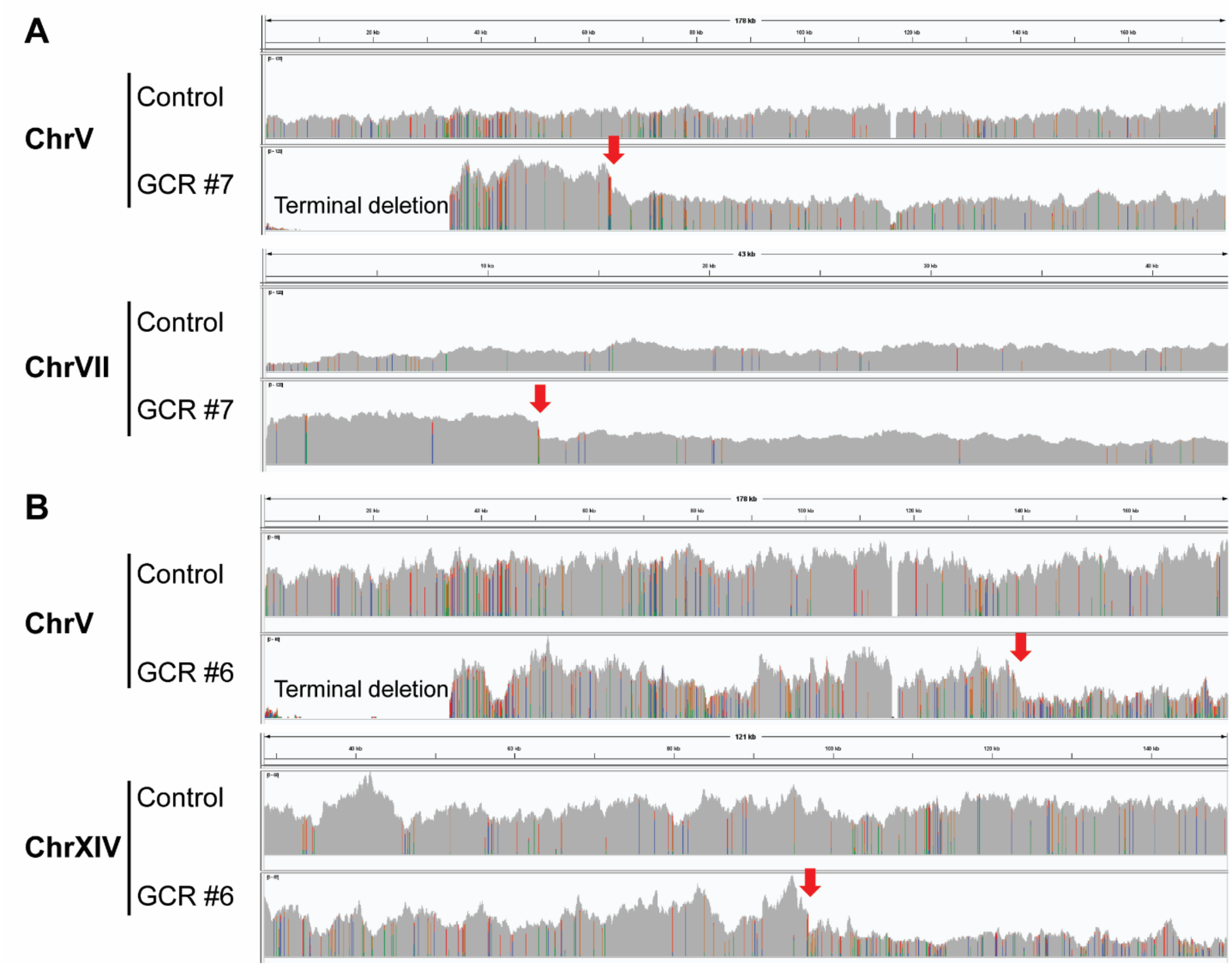


**Figure S4. Analysis of GCRs resulting from IR interaction in sub-telomeric ssDNA. Related to sequencing analysis.**

Twelve Can$^{R}$Ade$^{-}$ clones derived from *cdc13-707fs* harboring a 1.5 kb spaced *Alu*-IR were whole-genome, nanopore sequenced and reads were aligned to the *S. cerevisiae* reference genome. Terminal deletions of ChrV are indicated and translocation breakpoints are highlighted with a red arrow and lie adjacent to a duplicated region. **A.** An example of a GCR involving a ~ 24 kb duplication of ChrV with a breakpoint on ChrV at *PAU2* and invasion into a subtelomeric *PAU* gene (8/12 clones). **B.** An example of a GCR involving a ~ 100 kb duplication of ChrV with a breakpoint near a *Ty1* element or tRNA gene with invasion into either a *Ty1* element or tRNA gene, respectively (4/12 clones).

**Table S1. Mutagenesis in *cdc13-707fs* is mainly dependent on Rev3. Related to Figure 1.**

| Genetic background | Mutation rate in *CAN1* (X $10^{-7}$) |
|---|---|
| WT | 2.0 (1.8-2.7) |
| *rev3*Δ | 1.8 (1.1-3.0) |
| *cdc13-707fs* | 20.5 (16.1-35.0) |
| *cdc13-707fsrev3*Δ | 4.1 (2.9-5.5) |

**Table S2. Mutational spectrum of UV-induced $Can^R Lys^-$ clones in *cdc13-707fs.* Related to Figure 1.**

| WT base (in un-resected strand) | Mutation | *can1* | *lys2* | *trp1* | *mutation in dsDNA/total* | |
|---|---|---|---|---|---|---|
| A | G | | | 1 | AT to GC | Simple base substitutions |
| | T | 1 | 2 | | AT to TA | |
| | C | | 1 | | AT to CG | |
| **Total A** | | 1 | 3 | 1 | 5 | |
| G | A | | 2 | | GC to AT | |
| | C | 1 | 1 | | GC to CG | |
| | T | | 5 | | GC to TA | |
| Total G | | 1 | 8 | 0 | 9 | |
| PuPu context | | 1 | 7 | 1 | | |
| Total Pu | | 2 | 11 | 1 | | |
| T | C | 13 | 11 | | TA to CG | |
| | A | 9 | 11 | 1 | TA to AT | |
| | G | 1 | 1 | | TA to GC | |
| Total T | | 23 | 23 | 1 | 47 | |
| C | T | 4 | 7 | 1 | CG to TA | |
| | G | | | 1 | CG to GC | |
| | A | | 1 | | CG to AT | |
| Total C | | 4 | 8 | 2 | 14 | |
| PyPy context | | 25 | 27 | 2 | | |
| Total Py | | 27 | 31 | 3 | | |
| Total substitutions | | 29 | 42 | 4 | 75 | |
| Indel in a run of A | | | 1 | | 1 | Simple indels[1] |
| Other A indels | | | | | | |
| Total single A indel | | 0 | 1 | | 1 | |
| Indel in a run of G | | 1 | | | 1 | |
| Other G indels | | | | | | |
| Total single G indel | | 1 | 0 | | 1 | |
| Indel in a run of T | | 6 | 8 | | 14 | |
| Other T indels | | 4 | 1 | | 5 | |
| Total single T indel | | 10 | 9 | | 19 | |
| Indel in a run of C | | | 1 | | 1 | |
| Other C indels | | | 3 | | 3 | |
| Total single C indel | | 0 | 4 | | 4 | |
| Other indels | | 5 | 5 | | 12 | |
| Total indels[2] | | 16 | 19 | | 37 | |

[1] Indel mutations that deleted or inserted base(s) can be assigned to a homo-nucleotide or non-homonucleotide runs.

[2] Includes all simple indel mutations, regardless of the presence of a homo-nucleotide run.

**Table S3. Effect of *EXO1* deletion on GCRs induced by *Alu*-IRs with 200bp spacer in *cdc13-707fs.* Related to Figure 3.**

| Strain | GCR rate ($X 10^{-6}$) |
|---|---|
| wild-type | 1.8 (1.7-2.0) |
| *exo1Δ* | 2.6 (2.1-2.7) |
| *cdc13-707fs* | 178.6 (116.7-222.6) |
| *cdc13-707fsexo1Δ* | 10.2 (7.1-13.7) |

**Table S4. Structural analysis of *Alu*-IR-mediated GCRs in *cdc13-707fs*. Related to sequencing analysis.**

| # | Barcode | ChrV breakpoint | | Translocation breakpoint |
|---|---|---|---|---|
| GCR1 | 17 | 138,554 | YELWdelta6 | ChrXIV 96,942 YNLCdelta1 (Ty1) |
| GCR2 | 18 | 64,106 | near PAU2 | ChrXV 11,960 near PAU20 |
| GCR3 | 19 | 64,095 | near PAU2 | ChrXII 1,062,871 near PAU4 |
| GCR4 | 20 | 135,945 | YARWdelta6 (Ty1) (arm of ChrV recombined with ChrXIV at 777,126) | ChrI 183,137 near YELCdelta4 (Ty1) |
| GCR5 | 21 | 64,192 | near PAU2 | ChrVII 1,080,186 YGR293C near PAU12 |
| GCR6 | 22 | 135,501 | near YNCE0009C (lysine tRNA) | ChrXI 203,079 near YNCK0007W (lysine tRNA) |
| GCR7 | 23 | 64,180 | near PAU2 | ChrVII 12,344 near PAU13 |
| GCR8 | 24 | 64,090 | near PAU2 | ChrXII 13,484 near PAU18 |
| GCR9 | 25 | 64,106 | near PAU2 | ChrII 8,000 near PAU9 |
| GCR10 | 26 | 135,945 | near YELCdelta4 | ChrIII 169,207 near YCRWdelta11 (Ty1) |
| GCR11 | 27 | 64,091 | near PAU2 | ChrXV 11,947 near PAU20 |
| GCR12 | 28 | 64,106 | near PAU2 | ChrII 8,007 near PAU9 |

**Table S6. Strains used in this study.**

| Strain number | *Genotype* | Comment/Reference |
|---|---|---|
| **The strains below are in the following genetic background:** ***MATa, bar1Δ, his7-2, trp1Δ, ura3Δ, leu2-3,112, ade2Δ, lys2Δ, cup1Δ, yhr054cΔ, cup2Δ, V34205::ADE2LYS2, V29616::CUP1*** | | |
| **YNS191, YNS192** | *LYS2* | Zhang et al., 2012 |
| **YKL3477, YKL3478** | *LYS2, cdc13-707fs:URA3* | This study |
| **YKL5884, YKL5888** | *lys2::65% Alu-IR12bp* | This study |
| **YKL5892, YKL5896** | *lys2::65% Alu-IR12bp, cdc13-707fs:URA3* | This study |
| **YKL3736, YKL3737** | *lys2::75% Alu-IR12bp* | This study |
| **YKL3754, YKL3759** | *lys2::75% Alu-IR12bp, cdc13-707fs:URA3* | This study |
| **YKL3734, YKL3735** | *lys2::86% Alu-IR12bp* | This study |
| **YKL3747, YKL3750** | *lys2::86% Alu-IR12bp, cdc13-707fs:URA3* | This study |
| **KT21** | *lys2::94% Alu-IR12bp* | This study |
| **YKL3107, YKL3353** | *lys2::94% Alu-IR12bp, cdc13-707fs:URA3* | This study |
| **KT19, KT20** | *lys2::100% Alu-IR12bp* | Naraynan et al., 2006 |
| **YKL3270, YKL3271** | *lys2::100% Alu-IR12bp, cdc13-707fs:URA3* | This study |
| **YKL3806, YKL3807** | *lys2::100% Alu-IR200bp* | This study |
| **YKL3842, YKL3843** | *lys2::100% Alu-IR200bp, cdc13- 707fs:URA3* | This study |
| **YKL383, YKL384** | *lys2::100% Alu-IR1.5kb* | This study |
| **YKL3899, YKL3901** | *lys2::100% Alu-IR1.5kb, cdc13-707fs:URA3* | This study |
| **KT849, KT850** | *lys2::100% Alu-IR3.2kb* | This study |
| **YKL3905, YKL3909** | *lys2::100% Alu-IR3.2kb, cdc13-707fs:URA3* | This study |
| **YKL4110, YKL4111** | *lys2::100% Alu-IR5kb* | This study |
| **YKL4188, YKL4191** | *lys2::100% Alu-IR5kb, cdc13-707fs:URA3* | This study |
| **YKL4112, YKL4113** | *lys2::100% Alu-IR7kb* | This study |
| **YKL4196, YKL4198** | *lys2::100% Alu-IR7kb, cdc13-707fs:URA3* | This study |
| **YKL4114, YKL4115** | *lys2::100% Alu-IR10kb* | This study |
| **YKL4202, YKL4205** | *lys2::100% Alu-IR10kb, cdc13-707fs:URA3* | This study |
| **YNS115, YNS116** | *LYS2, rev3::kanMX* | This study |

| | | |
|---|---|---|
| **YKL3613, YKL3614** | *LYS2, cdc13-707fs:URA3, rev3::kanMX* | This study |
| **YKL3932, YKL3933** | *lys2::100% Alu-IR200bp, exo1::kanMX* | This study |
| **YKL4134, YKL4135** | *lys2::100% Alu-IR200bp, cdc13-707fs:URA3, exo1::kanMX* | This study |
| **YKL4742, YKL4743** | *lys2::100% Alu-IR1.5kb, rad1::natMX* | This study |
| **YKL4784, YKL4786** | *lys2::100% Alu-IR1.5kb, cdc13- 707fs, rad1::natMX* | This study |
| **YKL4744, YKL4745** | *lys2::100% Alu-IR1.5kb, slx4:: NatMX* | This study |
| **YKL4787, YKL4788** | *lys2::100% Alu-IR1.5kb, cdc13-707fs:URA3, slx4::natMX* | This study |
| **YKL5130, YKL5131** | *lys2::100% Alu-IR1.5kb, rad52::TRP1* | This study |
| **YKL5097, YKL5098** | *lys2::100% Alu-IR1.5kb, cdc13- 707fs, rad52::TRP1* | This study |
| **YKL5133, YKL5134** | *lys2::100% Alu-IR1.5kb, rad59::hphMX* | This study |
| **YKL5136, YKL5140** | *lys2::100% Alu-IR1.5kb, cdc13- 707fs, rad59::hphMX* | This study |
| **YKL3517, YKL3518** | *cdc13-1* | This study |
| **KT1924** | *LYS2 DDC1-yEGFP:KanMX* | This study |
| **KT1928, KT1929** | *LYS2 DDC1-yEGFP:KanMX cdc13-707fs:URA3* | This study |
| **YKL3948, YKL3949** | *V34205::ADE2:TRP1:LYS2* | *ADE2-TRP1-LYS2 Cassette.* This study |
| **YKL4094, YKL4096** | *V34205::ADE2:TRP1:LYS2, cdc13-707fs:URA3* | |
| **The strains below are in the following genetic background:** ***MATa, bar1Δ, his7-2, trp1Δ, ura3Δ, leu2-3,112, ade2Δ, lys2Δ, cup1Δ, yhr054cΔ, cup2Δ, V34205::ADE2LYS2, V29616::CUP1, can1::TRP1*** | | |
| **YKL2907, YKL2908** | *V47710::CAN1:natMX* | *CAN1NatMX cassette 48kb from telomere.* This study |
| **YKL3464, YKL3484** | *V47710::CAN1:natMX, cdc13-707fs:URA3* | |
| **YKL2909, YKL2910** | *V53745::CAN1:natMX* | *CAN1NatMX cassette 55kb from telomere.* This study |
| **YKL3466, YKL3467** | *V53745::CAN1:natMX, cdc13-707fs:URA3* | |
| **YKL3460, YKL3461** | *V71976::CAN1:natMX* | *CAN1NatMX cassette 73kb from telomere.* This study |
| **YKL3465, YKL3488** | *V71976::CAN1:natMX, cdc13-707fs:URA3* | |
| **YKL5042, YKL5043** | *V92341::CAN1:natMX* | *CAN1NatMX cassette 92kb from telomere.* This study |
| **YKL5064, YKL5067** | *V92341::CAN1:natMX, cdc13-707fs:URA3* | |

| The strains below the following genetic background:<br>MATa, *his7-2, leu2-3,112, trp1Δ, ura3Δ, lys2Δ, ade2Δ, bar1Δ, sfa1Δ, cup1-1Δ, yhr054cΔ, cup1-2Δ, can1-Δ* | | |
|---|---|---|
| **YKL5152, YKL5153** | *V7981::CAN1:ADE2, V38477::LYS2::kanMX:Alu, V32231::Alu:URA3* | 10 kb spacer *Alu*-IR (100% homology). This study |
| **YKL5270, YKL5271** | *V7981::CAN1:ADE2, V38477::LYS2::kanMX:Alu, V32231::Alu:URA3, cdc13-707fs:TRP1* | |
| **YKL5157, YKL5158** | *V7981::CAN1:ADE2, V38477::LYS2::kanMX:Alu, V23351:: Alu:URA3* | 20 kb spacer *Alu*-IR (100% homology). This study |
| **YKL5272, YKL5273** | *V7981::CAN1:ADE2, V38477::LYS2::kanMX:Alu, V23351:: Alu:URA3, cdc13-707fs:TRP1* | |
| **YKL5162, YKL5163** | *V7981::CAN1ADE2, V38477::LYS2::kanMX:Alu, V13095:: Alu:URA3* | 20 kb spacer *Alu*-IR (100% homology). This study |
| **YKL5276, YKL5277** | *V7981::CAN1ADE2, V38477::LYS2::kanMX:Alu, V13095:: Alu:URA3, cdc13-707fs:TRP1* | |

## Table S7. Oligonucleotides used in this study.

| Name | Sequence (5’ to 3‘) | Description |
|---|---|---|
| **Y’-F** | CGCGAATTCGCCCTACAGCACTTCTAC ATAGC | Y'-element fragment for telomere length measurement |
| **Y’-R** | CGAGAATTCCAGCGTTTGCGTTCCATGACG | |
| **S3630** | CTTCTACTCTTGACACTGAATACTAC | *LYS2* specific probe for inverted dicentric dimer detection |
| **O3970** | GGGAGGATTCAAAGACGTAGCAACA | |
| **seqDG_101** | CGTGGGCAAACACTTTGA | *LYS2* sequencing primer |
| **seqDG_103** | CCATAACCACAATCAAGG | |
| **seqDG_106** | TGCAACCATACTTACTCA | |
| **seqDG_108** | CCACACCCCTAGAAGAAT | |
| **seqDG_111** | TGACTAACGAAAAGGTCT | |
| **seqDG_113** | CGCTAAGCGATCCATCCA | |
| **seqDG_115** | ACAAAGACACCAGAACAG | |
| **seqDG_116** | GTAAGTATGGTTGCACAG | |
| **seqDG_117** | AGCTACTAGTTGTTAACA | |
| **seqDG_118** | AAGTTAAAATTCGTGGGT | |

| | | |
|---|---|---|
| **seqDG_120** | GCACAATTTTCAAGTATC | |
| **oKC109new** | GTAAGCTGCTGCGGAGCT | |
| **seqDG_94** | AGGATACAACGCATTTTC | |
| **seqDG_99** | CGAAAATTCCGTGAATAT | |
| **ADE2-3end** | GTTATGATTACATCAAATGTGG | *TRP1* sequencing primer |
| **LYS2-5end** | AGACCTTTTCGTTAGTCATT | |
| **oKC062** | AGGGTGAGAATGCGAAATG | *CAN1* sequencing primer |
| **seqDG_83** | CAAATTCAAAAGAAGACG | |
| **seqDG_84** | ACGCAGTCCTTGGGTGAA | |
| **seqDG_86** | TTGGTCTATCAAAGAACA | |
| **seqDG_89** | AACTCGTCACGAGAGATG | |
| **seqDG_91** | TTTGACAGGGAACAAGTT | |
| **SRI1-t2** | GCTGAGAAGCTGGTAAGTTGT | |
